# Supplementary figures and images for: Cooperative environmental engineering via biofilm formation can stabilize consumer-resource systems
Source: PLoS One. 2025 Dec 8;20(12):e0337943. doi: 10.1371/journal.pone.0337943 (PMC12685189; doi:10.1371/journal.pone.0337943)

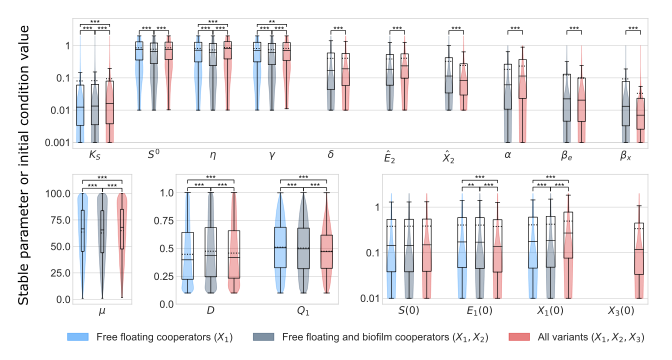

Supplement: S1 Fig — Box plot with the average shown in a dotted line overlays a violin plot of the distribution of stable parameter and initial condition values for the cases where only free floating cooperators are present (light blue), both free floating and biofilm cooperators are present (dark blue), and all variants (both cooperators plus the cheater) are present (red). Parameters or initial conditions randomly selected from a log-uniform distribution have a log Y-axis, and those selected from a uniform distribution have a linear Y-axis. A pairwise two-sided Mann-Whitney U test was for each parameter or initial condition, and the P value is denoted in text above the box/violin plots as , **, *** for P values P < .05, P < .01, and P < .001 respectively. For explicit P values see S2 Table and for mean and IQR values see S3 Table. (TIFF) [file pone.0337943.s001.tiff]

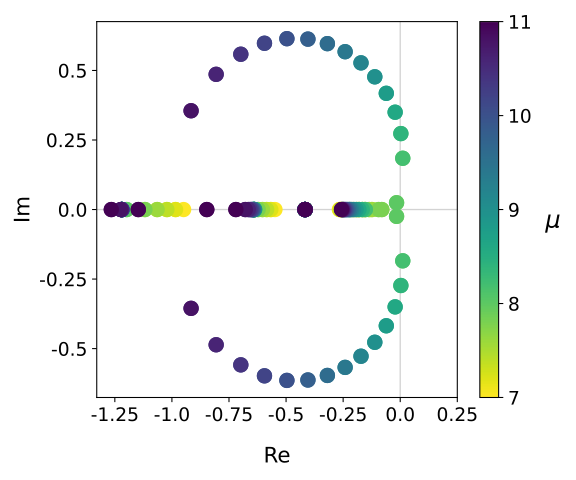

Supplement: S2 Fig — Scatter plot showing the real (X-axis) and imaginary (Y-axis) parts of the Eigenvalues as the maximum growth rate, μ, increases from 7 to 9 as indicated by the color of the marker. A pair of eigenvalues crosses the imaginary axis where the stable cycles were observed (Fig 4 main text, top), near μ=8.0, indicating a Hopf bifurcation. (TIFF) [file pone.0337943.s002.tiff]

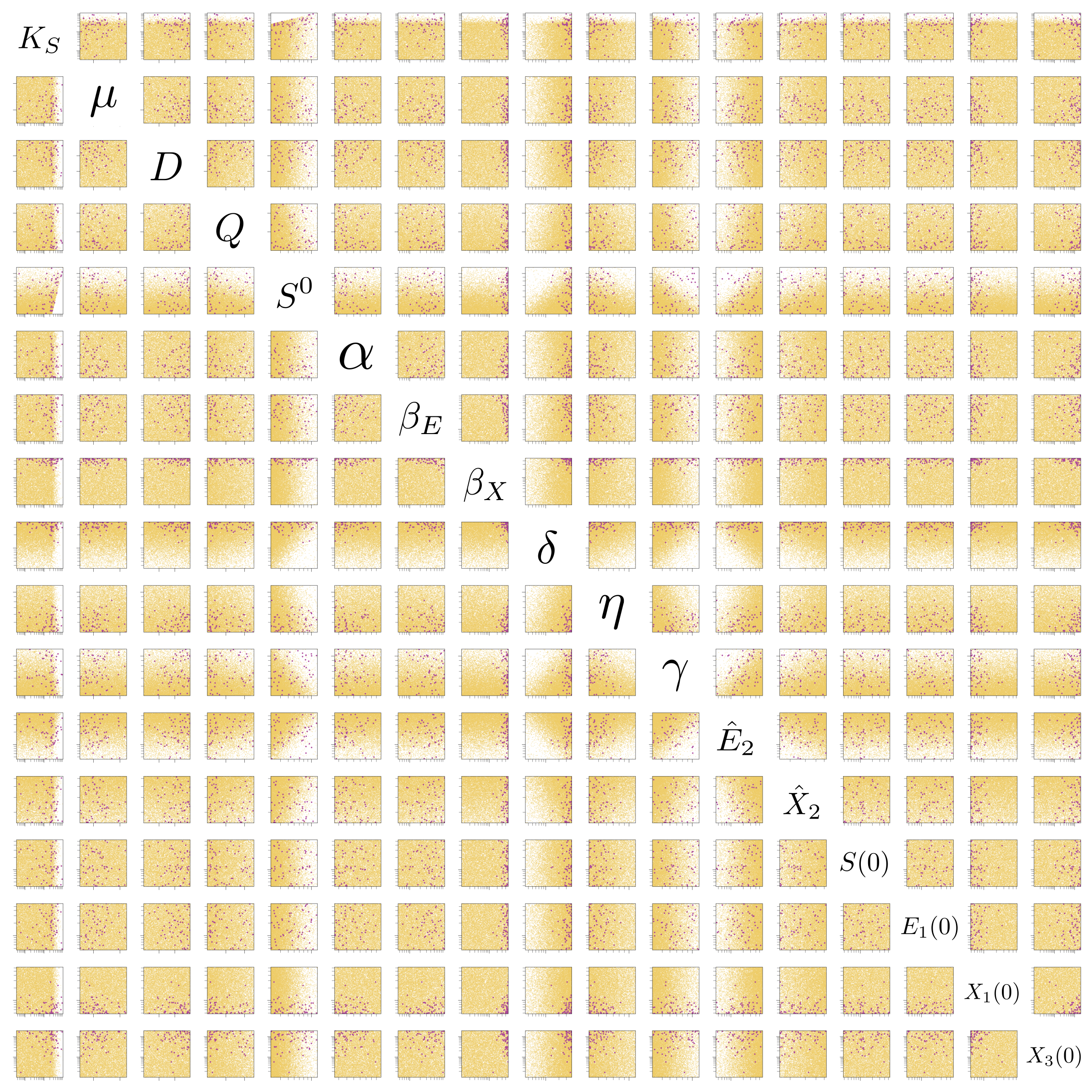

Supplement: S3 Fig — Figure components are the same as described in Fig 4 of the main text. (TIFF) [file pone.0337943.s003.tiff]

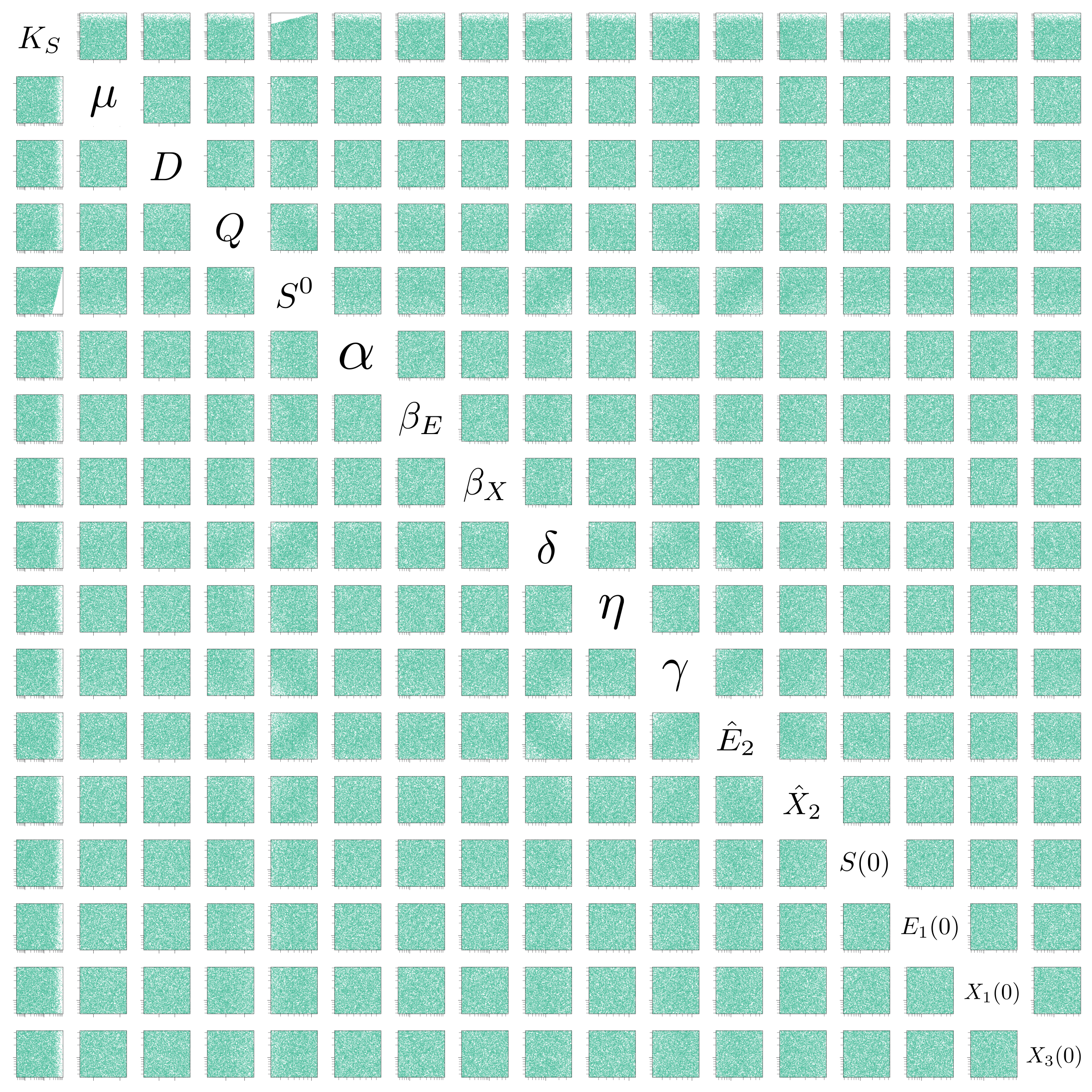

Supplement: S4 Fig — Each point represents a single simulation that resulted in full system stability. Graph labels are indicated across the diagonal. Full stable coexistence occurred across a wide range of values. (TIFF) [file pone.0337943.s004.tiff]
