# Supplementary material for: Cooperative environmental engineering via biofilm formation can stabilize consumer-resource systems
Source: PLoS One. 2025 Dec 8;20(12):e0337943. doi: 10.1371/journal.pone.0337943 (PMC12685189; doi:10.1371/journal.pone.0337943)
Supplement: S1 Table — (DOCX) [file pone.0337943.s005.docx]

Table S1: Model parameter and variable descriptions

| **Parameter or variable** | **Biological interpretation** | **Units** |
| --- | --- | --- |
| *S,*  *S^0^* | Nutrient substrate concentration,  Nutrient concentration entering the chemostat | mass/volume |
| *E_1_* | Free floating public good enzyme | mass/volume |
| *E_2_* | Public good enzyme in biofilm | mass/area |
| *Ê_2_* | Maximum amount of enzyme the biofilm can hold | mass/area |
| $M\left( E_{2} \right)=E_{2}/ \hat{E}_{2}$ | Fraction of occupied space for enzyme in the biofilm | - |
| *X_1_* | Free floating cooperating bacteria in fluid culture | gDW/volume |
| *X_2_* | Cooperating bacteria in the biofilm | gDW/area |
| $\hat{X}_{2}$ | Maximum amount of bacteria the biofilm can hold | gDW/area |
| $W\left( X_{2} \right)=X_{2}/\hat{X}_{2}$ | Fraction of occupied space for bacteria in the biofilm | - |
| *X_3_* | Free floating cheating bacteria in liquid bioreactor culture | gDW/area |
| $F\left( S,E_{i} \right)=\frac{\mu\cdot S\cdot E_{i}}{K_{S}+S}$ | Function for bacterial growth rate | 1/time |
| $\mu$ | Maximum growth rate | - |
| K_S_ | Nutrient half-saturation constant for growth function | mass/volume |
| α | Rate of bacterial wall adhesion | 1/time |
| β_X_ | Rate of bacterial wall sloughing | 1/time |
| β­_E_ | Rate of enzyme wall sloughing | 1/time |
| *D* | Dilution rate | 1/time |
| Q | Fraction of nutrient uptake devoted to quorum sensing behaviors | - |
| η | Enzyme produced per bacterial biomass | mass/gDW |
| γ | Conversion of nutrient to biomass | gDW/mass |
| δ | Bacterial biomass and enzyme concentration conversion from area to volume | $\frac{gDW/volume}{gDW/area}=\frac{\mathrm{area}}{\mathrm{volume}}$ |
