# Supplementary material for: Cooperative environmental engineering via biofilm formation can stabilize consumer-resource systems
Source: PLoS One. 2025 Dec 8;20(12):e0337943. doi: 10.1371/journal.pone.0337943 (PMC12685189; doi:10.1371/journal.pone.0337943)
Supplement: S2 Table — (DOCX) [file pone.0337943.s006.docx]

Table S2: *P* values for Mann Whitney U test comparing stable parameter and initial condition values from S1 Fig.

| **Parameter or  initial condition** | ***P value for pairwise combinations*** | | |
| --- | --- | --- | --- |
|  | **X_1_**  **v**  **X_1_ and X_2_** | **X_1_**  **v**  **All variants** | **X1 and X2**  **v**  **All variants** |
| *K­_S_* | 1.41E-15 | 7.53E-35 | 5.67E-20 |
| *S^0^* | 0 | 0.544636 | 1.87E-87 |
| δ |  |  | 2.00E-90 |
| η | 0 | 4.37E-44 | 1.27E-231 |
| γ | 5.69E-287 | 0.005219 | 6.08E-74 |
| *Ê_2_* | 1.05E-103 | 6.93E-74 | 6.15E-19 |
| $\hat{X}_{2}$ | 0 | 1.41E-287 | 1.73E-100 |
| α |  |  | 5.38E-205 |
| β­_E_ |  |  | 3.05E-12 |
| β_X_ |  |  | 0 |
| µ | 2.69E-22 | 3.45E-10 | 9.28E-25 |
| *D* | 2.90E-222 | 9.95E-09 | 6.71E-14 |
| Q | 1.96E-24 | 3.12E-79 | 1.76E-46 |
| $S(0)$ | 0.406178 | 0.096678 | 0.186409 |
| $E_{1}(0)$ | 0.001806 | 7.08E-31 | 1.49E-24 |
| $X_{1}\left( 0 \right)$ | 2.90E-13 | 2.92E-152 | 3.94E-119 |
| $X_{3}\left( 0 \right)$ |  |  | 0 |

Parameters or initial conditions that were absent in the case where the free floating cooperators are alone (*X*_1_ only) have a black fill as there was no test to run. All values indicated are as reported by the SciPy stats Mann-Whitney U test function.
