# Supplementary material for: Cooperative environmental engineering via biofilm formation can stabilize consumer-resource systems
Source: PLoS One. 2025 Dec 8;20(12):e0337943. doi: 10.1371/journal.pone.0337943 (PMC12685189; doi:10.1371/journal.pone.0337943)
Supplement: S3 Table — (DOCX) [file pone.0337943.s007.docx]

Table S3: Box plot statistics

| **Parameter or initial condition** | **Condition** | **Median** | **Mean** | **Q1** | **Q3** |
| --- | --- | --- | --- | --- | --- |
| ***K­_S_*** | X1 | 0.0124 | 0.0801 | 0.00336 | 0.0594 |
|  | X1 and X2 | 0.0134 | 0.0820 | 0.00352 | 0.0631 |
|  | All variants | 0.01617 | 0.0940 | 0.00377 | 0.0797 |
| ***S^0^*** | X1 | 0.766 | 0.853 | 0.358 | 1.309 |
|  | X1 and X2 | 0.653 | 0.772 | 0.278 | 1.206 |
|  | All variants | 0.762 | 0.852 | 0.372 | 1.284 |
| **δ** | X1 | NA | NA | NA | NA |
|  | X1 and X2 | 0.114 | 0.325 | 0.0340 | 0.421 |
|  | All variants | 0.0838 | 0.243 | 0.0294 | 0.274 |
| **η** | X1 | 0.720 | 0.817 | 0.313 | 1.272 |
|  | X1 and X2 | 0.603 | 0.736 | 0.240 | 1.164 |
|  | All variants | 0.807 | 0.876 | 0.386 | 1.333 |
| **γ** | X1 | 0.713 | 0.815 | 0.314 | 1.268 |
|  | X1 and X2 | 0.621 | 0.749 | 0.257 | 1.175 |
|  | All variants | 0.715 | 0.820 | 0.346 | 1.242 |
| ${\hat{\boldsymbol{E}}}_{\boldsymbol{2}}$ | X1 | NA | NA | NA | NA |
|  | X1 and X2 | 0.167 | 0.402 | 0.0436 | 0.591 |
|  | All variants | 0.191 | 0.405 | 0.0575 | 0.575 |
| ${\hat{\boldsymbol{X}}}_{\boldsymbol{2}}$ | X1 | NA | NA | NA | NA |
|  | X1 and X2 | 0.185 | 0.385 | 0.0593 | 0.530 |
|  | All variants | 0.236 | 0.406 | 0.0978 | 0.557 |
| **α** | X1 | NA | NA | NA | NA |
|  | X1 and X2 | 0.0609 | 0.184 | 0.0107 | 0.265 |
|  | All variants | 0.114 | 0.232 | 0.0232 | 0.3726 |
| **β­_E_** | X1 | NA | NA | NA | NA |
|  | X1 and X2 | 0.0224 | 0.122 | 0.00455 | 0.130 |
|  | All variants | 0.0204 | 0.0990 | 0.00444 | 0.0993 |
| **β_X_** | X1 | NA | NA | NA | NA |
|  | X1 and X2 | 0.0132 | 0.0929 | 0.00326 | 0.0775 |
|  | All variants | 0.00699 | 0.0332 | 0.00257 | 0.0233 |
| **µ** | X1 | 66.59 | 63.56 | 45.37 | 84.20 |
|  | X1 and X2 | 65.57 | 62.69 | 43.97 | 83.70 |
|  | All variants | 68.08 | 64.92 | 47.55 | 85.09 |
| ***D*** | X1 | 0.398 | 0.447 | 0.223 | 0.644 |
|  | X1 and X2 | 0.437 | 0.475 | 0.245 | 0.686 |
|  | All variants | 0.417 | 0.458 | 0.233 | 0.658 |
| **Q** | X1 | 0.506 | 0.511 | 0.326 | 0.690 |
|  | X1 and X2 | 0.496 | 0.503 | 0.318 | 0.682 |
|  | All variants | 0.470 | 0.474 | 0.322 | 0.619 |
| $\boldsymbol{S(0)}$ | X1 | 0.144 | 0.379 | 0.038 | 0.540 |
|  | X1 and X2 | 0.145 | 0.381 | 0.0381 | 0.542 |
|  | All variants | 0.149 | 0.384 | 0.0391 | 0.547 |
| $\boldsymbol{E}_{\boldsymbol{1}}\boldsymbol{(0)}$ | X1 | 0.172 | 0.405 | 0.0470 | 0.589 |
|  | X1 and X2 | 0.170 | 0.403 | 0.0450 | 0.589 |
|  | All variants | 0.138 | 0.375 | 0.0379 | 0.529 |
| $\boldsymbol{X}_{\boldsymbol{1}}\left( \boldsymbol{0} \right)$ | X1 | 0.175 | 0.408 | 0.0459 | 0.602 |
|  | X1 and X2 | 0.184 | 0.421 | 0.0480 | 0.629 |
|  | All variants | 0.270 | 0.494 | 0.0761 | 0.779 |
| $\boldsymbol{X}_{\boldsymbol{3}}\left( \boldsymbol{0} \right)$ | X1 | NA | NA | NA | NA |
|  | X1 and X2 | NA | NA | NA | NA |
|  | All variants | 0.117 | 0.337 | 0.0334 | 0.447 |
