# Supplementary material for: Cooperative environmental engineering via biofilm formation can stabilize consumer-resource systems
Source: PLoS One. 2025 Dec 8;20(12):e0337943. doi: 10.1371/journal.pone.0337943 (PMC12685189; doi:10.1371/journal.pone.0337943)
Supplement: S4 Table — (DOCX) [file pone.0337943.s008.docx]

**Table S4. Cooperator stable coexistence parameter ranges**

| **Parameter** | **Min** | **Max** | **Parameter** | **Min** | **Max** |
| --- | --- | --- | --- | --- | --- |
| **S^0^** | 0.3 | 1.2 | **α** | 0.15 | 0.3 |
| ${\hat{\boldsymbol{X}}}_{\boldsymbol{2}}$ | 0.15 | 0.53 | **β_X_** | 0.001 | 0.05 |
| $\boldsymbol{\mu}$ | 50 | 85 | **β­_E_** | *0.00455* | 0.1 |
| ***K_S_*** | *0.00352* | $S^{0}\cdot0.85$ | **η** | 0.4 | *1.164* |
| ***D*** | 0.2 | 0.5 | **γ** | 0.4 | *1.175* |
| **Q** | 0.4 | 0.65 | **δ** | *0.034* | 0.4 |

*Values in italics are set to the IQR value.*
