# Supplementary material for: Cooperative environmental engineering via biofilm formation can stabilize consumer-resource systems
Source: PLoS One. 2025 Dec 8;20(12):e0337943. doi: 10.1371/journal.pone.0337943 (PMC12685189; doi:10.1371/journal.pone.0337943)
Supplement: S1 File — (DOCX) [file pone.0337943.s009.docx]

## S1. Proof the model is well posed.

We show the model is well-posed in the sense that the system of equations in Figure 1 of the main text is dissipative. First, we can scale out the conversion factors γ, η, and δ by letting ${s=S, s^{0}=S^{0},e}_{1}=\frac{E_{1}}{\eta\cdot\gamma},$ $e_{2}=\frac{\delta\cdot E_{2}}{\eta\cdot\gamma}, x_{i}=\frac{X_{i}}{\gamma} \mathrm{for} i=1, 3, x_{2}=\frac{\delta\cdot X_{2}}{\gamma}, d=D, \mathrm{and} q=Q.$ Additionally, $f\left( s, e_{1} \right)=F\left( s,\gamma\cdot\eta\cdot e_{1} \right), f\left( s,e_{2} \right)=F\left( s,\frac{\gamma\cdot\eta}{\delta}\cdot e_{2} \right), W\left( x_{2} \right)=\frac{\gamma\cdot x_{2}}{\delta\cdot\hat{X}_{2}}$ and $W\left( e_{2} \right)=\frac{\eta\cdot\gamma\cdot e_{2}}{\delta\cdot\hat{E}_{2}}$. Note that the substitutions of *s, d,* and *q* are purely for aesthetic purposes. This leads to the following scaled model:

| substrate | $\dot{s}=d\cdot\left( s^{0}-s \right)-\left( \left( x_{1}+x_{3} \right)\cdot f\left( s,e_{1} \right)+x_{2}\cdot f(s,e_{2}) \right)$ | (1) |
| --- | --- | --- |
| free floating enzyme | $\dot{e}_{1}=q\cdot\left( x_{1}\cdot f\left( s,e_{1} \right)+M\left( e_{2} \right)\cdot x_{2}\cdot f\left( s,e_{2} \right) \right)+\beta_{E}\cdot e_{2}-d\cdot e_{1}$ | (2) |
| Biofilm enzyme | $\dot{e}_{2}=q\cdot x_{2}\cdot f\left( s,e_{2} \right)\cdot\left( 1-M\left( e_{2} \right) \right)-\beta_{E}\cdot e_{2}$ | (3) |
| Free floating cooperator | $\dot{x}_{1}=x_{1}\cdot\left( \left( 1-q \right)\cdot f\left( s,e_{1} \right)-\alpha\cdot\left( 1-W\left( x_{2} \right) \right)-d \right)$ | (4) |
|  | $+x_{2}\cdot\left( \beta_{X}+\left( 1-q \right)\cdot f\left( S,e_{2} \right)\cdot W(x_{2}) \right)$ | (5) |
| Biofilm cooperator | $\dot{x}_{2}=x_{2}\cdot\left( \left( 1-q \right)\cdot f\left( s,e_{2} \right)\cdot\left( 1-W\left( x_{2} \right) \right)-\beta_{X} \right)+\alpha\cdot x_{1}\cdot\left( 1-W\left( x_{2} \right) \right)$ | (6) |
| Free floating cheater | $\dot{x}_{3}=x_{3}\cdot\left( f\left( s,e_{1} \right)-d \right)$ | (7) |

**Lemma 1**. Assume that **N1** (main text) holds. All solutions of (1)-(7) forward invariant, meaning solutions initiated in $\mathbb{R}_{+}^{6}$ exist and remain in $\mathbb{R}_{+}^{6}$ for all $\tau>0$, and are bounded. System (1)-(7) is dissipative.

*Proof.*

Let $y=s+e_{1}+e_{2}+x_{1}+x_{2}+x_{3}$, then

$$\dot{y}=d\cdot\left( s^{0}+e_{2}+x_{2}-y \right)\leq d\cdot\left( s^{0}+\hat{E}_{2}+\hat{X}_{2}-y \right),$$

and hence the ${\lim\sup}_{\tau\to+\infty} y\left( \tau\right)\leq s^{0}+ \hat{E}_{2}+\hat{X}_{2},$ implying that (1)*-*(7) is bounded.

To show that all (1)*-*(7) is forward invariant requires setting each state variable to 0 and solving that variables equation. Doing so results in the following:

| substrate | $\dot{s}=d\cdot s^{0}>0$ | (8) |
| --- | --- | --- |
| free floating enzyme | $\dot{e}_{1}=q\cdot M\left( e_{2} \right)\cdot x_{2}\cdot f\left( s,e_{2} \right)+\beta_{E}\cdot e_{2}\geq0$ | (9) |
| Biofilm enzyme | $\dot{e}_{2}=0$ | (10) |
| Free floating cooperator | $\dot{x}_{1}=x_{2}\cdot\left( \beta_{X}+\left( 1-q \right)\cdot f\left( S,e_{2} \right)\cdot W\left( x_{2} \right) \right)\geq0$ | (11) |
| Biofilm cooperator | $\dot{x}_{2}=\alpha\cdot x_{1}\cdot\left( 1-W\left( x_{2} \right) \right)\geq0$ | (12) |
| Free floating cheater | $\dot{x}_{3}=0$ | (13) |

All equations in (8)-(13) are greater than or equal to zero, thus, all solution initiated in $\mathbb{R}_{+}^{6}$ will stay in $\mathbb{R}_{+}^{6}$. Hence, (8)-(13) are dissipative.

◻
