# Supplementary material for: Cooperative environmental engineering via biofilm formation can stabilize consumer-resource systems
Source: PLoS One. 2025 Dec 8;20(12):e0337943. doi: 10.1371/journal.pone.0337943 (PMC12685189; doi:10.1371/journal.pone.0337943)
Supplement: Attachment — Submitted filename: PONE-D-25-44591.docx [file pone.0337943.s010.docx]

This manuscript addresses the classic cooperation problem in evolutionary and microbial ecology by examining how cooperative behavior can persist in consumer–resource systems despite the threat of social cheating and the tragedy of the commons. The paper’s main contribution is modeling biofilm formation as a form of environmental engineering that helps stabilize cooperation. It also shows that environmental modification is a collective ecological process that is both biologically important and not yet well studied.

The article is well-written, however, I recommend the following revisions:

- The manuscript could more explicitly engage with the literature on partially privatized public goods (e.g., Solutions to the Public Goods Dilemma in Bacterial Biofilms by Drescher et al., 2014; Privatization of Biofilm Matrix in Structurally Heterogeneous Biofilms by Otto et al., 2020) and niche construction theory (e.g., Niche Construction Theory: A Practical Guide for Ecologists by Odling-Smee et al., 2013; An Introduction to Niche Construction Theory by Laland et al., 2016). Without this integration, the manuscript’s novelty may appear overstated.
- In lines 317–320, the authors introduce separate pools for enzymes in the fluid **(E1**) and biofilm **(E2)** but doesn’t fully justify this biologically.
- Lines 317–333 describe cooperators, enzymes, and flow processes in detail, much of which is later repeated in Table 1 (lines 363–364). This is similar to lines 345–352, which discuss the assumptions for uptake functions. Present concise definitions in the text, and direct readers to Table 1 for full biological interpretations and units, rather than repeating these twice.
- **Lines 270–277 (Discussion):** Link your findings to resilience theory, noting that biofilms provide structural feedbacks that increase system resilience by delaying or preventing collapse.
- **Lines 283–288 (Discussion):** The authors should broaden the interpretation by connecting microbial biofilm resilience to general principles in social-ecological systems (e.g., A General Framework for Analyzing Sustainability of Social-Ecological Systems By Ostrom 2009).
- The authors should conduct a careful proofread for typographical consistency and duplicate references (e.g., line 232 ‘liklihood’ → ‘likelihood’), remove duplicate or redundant references (e.g., line 582 and 588 Sandoz et al. 2007).
