# Supplementary material for: Cooperative environmental engineering via biofilm formation can stabilize consumer-resource systems
Source: PLoS One. 2025 Dec 8;20(12):e0337943. doi: 10.1371/journal.pone.0337943 (PMC12685189; doi:10.1371/journal.pone.0337943)
Supplement: Attachment — Submitted filename: Response to Reviewers.docx [file pone.0337943.s011.docx]

**Academic Editor:**

When submitting your revision, we need you to address these additional requirements.

1. Please ensure that your manuscript meets PLOS ONE's style requirements, including those for file naming. The PLOS ONE style templates can be found at

<https://journals.plos.org/plosone/s/file?id=wjVg/PLOSOne_formatting_sample_main_body.pdf> and

[https://journals.plos.org/plosone/s/file?id=ba62/PLOSOne_formatting_sample_title_authors_affiliations.pdf](https://track.editorialmanager.com/CL0/https:%2F%2Fjournals.plos.org%2Fplosone%2Fs%2Ffile%3Fid=ba62%2FPLOSOne_formatting_sample_title_authors_affiliations.pdf/1/010f0199710a2e54-8fbad2ac-d53d-472c-ba92-9320ef993d61-000000/YPKBQlJr-LPIXhgnx0q6iqA-0b2si3Ik8mLY5FIF7V4=229)

Necessary changes to file names and format have been completed to align with the PLOS ONE formatting style.

2. Thank you for uploading your study's underlying data set. Unfortunately, the repository you have noted in your Data Availability statement does not qualify as an acceptable data repository according to PLOS's standards.

At this time, please upload the minimal data set necessary to replicate your study's findings to a stable, public repository (such as figshare or Dryad) and provide us with the relevant URLs, DOIs, or accession numbers that may be used to access these data. For a list of recommended repositories and additional information on PLOS standards for data deposition, please see [https://journals.plos.org/plosone/s/recommended-repositories](https://track.editorialmanager.com/CL0/https:%2F%2Fjournals.plos.org%2Fplosone%2Fs%2Frecommended-repositories/1/010f0199710a2e54-8fbad2ac-d53d-472c-ba92-9320ef993d61-000000/VkLCDAJBQf7X4XCF-3P_VJ11HkYrtGoy6CcDTYjZZ9I=229).

We used Figshare as a repository, which is listed as an acceptable repository by PLOS ONE. We are unsure of what edits/changes need to be made here.

3. Please update your submission to use the PLOS LaTeX template. The template and more information on our requirements for LaTeX submissions can be found at [http://journals.plos.org/plosone/s/latex](http://track.editorialmanager.com/CL0/http:%2F%2Fjournals.plos.org%2Fplosone%2Fs%2Flatex/1/010f0199710a2e54-8fbad2ac-d53d-472c-ba92-9320ef993d61-000000/Dyqrj-PULnxWYf-C33SkaoVsjZ2JI3g30VzBsoE5Vhk=229).

We have updated our manuscript to be in a LaTeX file format.

4. If the reviewer comments include a recommendation to cite specific previously published works, please review and evaluate these publications to determine whether they are relevant and should be cited. There is no requirement to cite these works unless the editor has indicated otherwise. 

Please review your reference list to ensure that it is complete and correct. If you have cited papers that have been retracted, please include the rationale for doing so in the manuscript text, or remove these references and replace them with relevant current references. Any changes to the reference list should be mentioned in the rebuttal letter that accompanies your revised manuscript. If you need to cite a retracted article, indicate the article’s retracted status in the References list and also include a citation and full reference for the retraction notice.

We have added some citations that the reviewer recommended or were related to topics the reviewer suggested including.

**Reviewer 1:**
This manuscript addresses the classic cooperation problem in evolutionary and microbial ecology by examining how cooperative behavior can persist in consumer–resource systems despite the threat of social cheating and the tragedy of the commons. The paper’s main contribution is modeling biofilm formation as a form of environmental engineering that helps stabilize cooperation. It also shows that environmental modification is a collective ecological process that is both biologically important and not yet well studied.

We appreciate your careful review of our manuscript and for the highly constructive comments. We responded to each comment below.

The article is well-written, however, I recommend the following revisions:

• The manuscript could more explicitly engage with the literature on partially privatized public goods (e.g., Solutions to the Public Goods Dilemma in Bacterial Biofilms by Drescher et al., 2014; Privatization of Biofilm Matrix in Structurally Heterogeneous Biofilms by Otto et al., 2020) and niche construction theory (e.g., Niche Construction Theory: A Practical Guide for Ecologists by Odling-Smee et al., 2013; An Introduction to Niche Construction Theory by Laland et al., 2016). Without this integration, the manuscript’s novelty may appear overstated.

Thank you for the suggestion. We agreed that adding more discussion on these topics enhances the quality of the manuscript and arguments made within it. We’ve included additional text in the introduction expanding on partially public goods (lines 79-89) and how environmental engineering and Niche Construction Theory are related (lines 30-38). We also added text into the discussion highlighting how the results of Otto et al. 2020 relate to our results (lines 251-265).

• In lines 317–320 (now 298-299), the authors introduce separate pools for enzymes in the fluid (E1) and biofilm (E2) but doesn’t fully justify this biologically.

We agree that the rationale we included could have been more clear, and we have rewritten our justification (lines 299-301).

• Lines 317–333 (now 297-310), describe cooperators, enzymes, and flow processes in detail, much of which is later repeated in Table 1 (lines 363–364). This is similar to lines 345–352 (now 329-344), which discuss the assumptions for uptake functions. Present concise definitions in the text, and direct readers to Table 1 for full biological interpretations and units, rather than repeating these twice.

Thank you for pointing out the redundant text. The table was merely meant to be an easy reference guide for readers while parsing the text, as such, we have moved the table into supplemental materials (S1 Table) to reduce redundancy.

• Lines 270–277 (now 209-217) (Discussion): Link your findings to resilience theory, noting that biofilms provide structural feedbacks that increase system resilience by delaying or preventing collapse.

We agree that a more explicit connection to resilience theory is warranted. Lines 224-227 have been added to more directly link our results to resilience theory.

• Lines 283–288 (now 222-232) (Discussion): The authors should broaden the interpretation by connecting microbial biofilm resilience to general principles in social-ecological systems (e.g., A General Framework for Analyzing Sustainability of Social-Ecological Systems By Ostrom 2009).

Thank you for this compelling idea to strengthen our discussion. We have added a paragraph linking our system to Social-Ecological System Framework in lines 233-250.

• The authors should conduct a careful proofread for typographical consistency and duplicate references (e.g., line 232 ‘liklihood’ → ‘likelihood’), remove duplicate or redundant references (e.g., line 582 and 588 Sandoz et al. 2007).

Thank you for bringing these errors to our attention. A careful revision of the bibliography and text was done to resolve typographical errors.
